# Analysis of the exercise intention-behavior gap among college students using explainable machine learning

**DOI:** 10.3389/fpubh.2025.1613553

**Published:** 2025-07-25

**Authors:** Cui Cui, Jixin Yin

**Affiliations:** ^1^Department of Sports, Huanghe Jiaotong University, Jiaozuo, Henan, China; ^2^Gangneung Wonju University, Gangneung, Gangwon Province, Republic of Korea

**Keywords:** physical activity promotion, college student, intention-behavior gap, explainable machine learning, feature engineering

## Abstract

**Introduction:**

The physical fitness of college students is a growing global public health concern. A critical challenge in improving student fitness is addressing the intention-behavior gap–the disconnect between students' intentions to engage in physical activity and their actual behavior.

**Methods:**

This study utilized survey data from TikTok-using college students, incorporating variables such as gender, academic grade, health belief perceptions, and planned behavior perceptions. Multiple machine learning models were developed to predict the presence of the intention-behavior gap. The performance of these models was evaluated, and SHapley Additive exPlanations (SHAP) was applied to the best-performing model to interpret feature importance.

**Results:**

Among the models tested, SHAP analysis revealed that perceived barriers were the most influential factor contributing to the intention-behavior gap. Furthermore, the results indicated that male students in higher academic grades, with fewer perceived barriers and stronger subjective norms regarding physical activity, were significantly less likely to exhibit this gap.

**Discussion:**

These findings suggest that university health promotion strategies should focus on reducing perceived barriers, cultivating a supportive campus environment for physical activity, and optimizing the allocation of physical education resources. Such measures may effectively support the transformation of students' physical activity intentions into consistent, health-promoting behaviors.

## 1 Introduction

College students represent the backbone of a nation's future development, and their physical health is closely linked to the quality and sustainability of national human resources (1–3). A physically fit student population contributes not only to individual well-being but also to the long-term economic and social vitality of a country. However, a growing body of research has recently highlighted a continuous decline in their physical fitness, with various studies reporting alarming trends in reduced cardiovascular endurance, muscular strength, and flexibility among this population (4, 5). Among the various interventions proposed, physical exercise is widely recognized by scholars as an effective measure. Physical exercise improves one's lifestyle as well as promotes both physical and mental health in the long term, which plays a crucial role in building healthy habits that extend into adulthood (6, 7).

Although governments and universities around the world have implemented various policies to promote physical activity among college students, their actual participation rates remain relatively low (8). Moreover, the frequency, intensity, and consistency of students physical exercise often do not meet the recommended health guidelines (9). This phenomenon not only hinders the physical and mental well-being of college students but also, to some extent, restricts the development of physical education in higher education institutions. Therefore, it is essential to effectively foster their initiative and persistence in physical exercise.

Currently, a key challenge in promoting college students' active participation in physical exercise lies in the significant gap between their intentions and actual behaviors. This discrepancy is known as the intention-behavior gap (10, 11). Even when individuals have a strong willingness to exercise, various influencing factors often hinder them from translating that intention into actual physical activity. The intention-behavior gap has been confirmed to be a common phenomenon in health intervention practices. For example, Wee et al. (12) explored how reducing the intention-behavior gap promotes an increase in physical exercise behavior. Wang et al. (13) indicated that the intention-behavior gap can influence sustained physical activity.

Many scholars have conducted examinations on mediating or moderating variables between exercise intention and behavior by drawing on the foundational frameworks of classic theories such as the Health Belief Model and the Theory of Planned Behavior (14, 15). However, the factors contributing to the intention-behavior gap in exercise are diverse, and existing research lacks a comprehensive analysis from a multi-variable, holistic perspective. Recently, the continuous advancement of artificial intelligence technologies has equipped scientific research with a diverse array of efficient and powerful analytical tools (16, 17). SHapley Additive exPlanation (SHAP)-based interpretable machine learning offers the capability to simultaneously process multiple feature variables while identifying those that exert the most significant influence on outcomes (18–20). Thus, this approach is particularly well-suited for holistically analyzing the predictive effects of various feature variables on the intention-behavior gap in college students' physical exercise.

Based on the above analysis and discussion, this study systematically incorporates multiple feature variables–health belief and planned behavior—to construct a computational model for predicting the intention-behavior gap in college students' physical exercise. Specifically, this study utilizes questionnaire data collected from TikTok to identify the optimal predictive model among Logistic Regression (LR) (21), Support Vector Machine (SVM) (22), and Random Forest (RF) (23). Subsequently, the SHAP method is employed to rank feature importance within the optimal model. From a multivariate and holistic perspective, a systematic analysis is conducted on the intention-behavior gap in college students physical exercise, which aims to provide evidence-based recommendations for promoting physical activity and enhancing the physical and mental well-being of university students. The main contributions of our work can be listed as follows:

1. We systematically identify and quantify key psychological and social factors that contribute to the exercise intention-behavior gap among college students by leveraging a large-scale dataset collected from TikTok, which provides empirical evidence in a novel digital social context.
2. We develop an interpretable machine learning framework that not only predicts the intention-behavior gap but also explains the underlying decision mechanisms, advancing the application of explainable AI in health behavior research.
3. We propose a decision tree-based pathway analysis that reveals actionable intervention targets. This offers practical guidance for designing personalized university sports programs and policies aimed at effectively narrowing the exercise intention-behavior gap.

The remainder of this paper is organized as follows: Section 2 presents theoretical analysis of exercise intention-behavior gap in college students. Section 3 describes preliminaries about machine learning evaluation metrics and SHAP explanation. Section 4 presents the data sources and preprocessing methods, and introduces a comprehensive framework based on interpretable machine learning. Section 5 conducts a systematic analysis of the exercise intention-behavior gap among college students based on explainable machine learning methods. Section 6 summarizes the entire study and, based on the research findings, provides corresponding recommendations for addressing the exercise intention-behavior gap among college students. Section 7 discusses the limitations of this study and the directions for future improvements.

## 2 Theoretical analysis of exercise intention-behavior gap in college students

**Figure 1:**
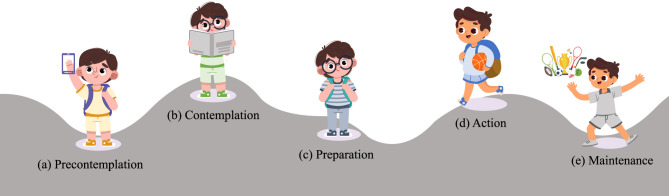
Five stages of intention-behavior transformation.

Overall, the research on the intention-behavior gap continues and expands upon traditional intention-behavior studies, still guided by the classical intention-behavior theoretical paradigm. In social psychology and health promotion, which extend from psychological theory, intention-behavior research contains two basic assumptions (24, 25). First, behavior is driven by behavioral intention. Second, most human behaviors are goal-oriented and exhibit rationality. In the context of university students' exercise behavior, this theoretical assumption reveals the gap between intention and actual behavior (26). University students' physical exercise behaviors generally go through a process of development from none to some and from weak to strong. Specifically, the formation of exercise behavior can be divided into five stages, as shown in Figure 1. When an individual's behavior remains at the first three stages and fails to transition into actual action or maintenance stages, the intention-behavior gap occurs. This is characterized by the presence of an intention to exercise but a lack of sustained actual behavior.

### 2.1 The perspective of health belief model

The health belief model, as a widely recognized theoretical framework for understanding individual health-related behaviors, posits that the adoption of such behaviors is largely influenced by an individual's subjective perceptions of health threats and their evaluations of coping strategies (27). The model comprises several core dimensions, including perceived benefits, perceived barriers, perceived severity, perceived susceptibility, and self-efficacy (28). In the context of physical activity, individuals who believe that exercise can effectively prevent illness and promote health, and who feel confident in their ability to overcome exercise-related barriers, are more likely to initiate and sustain regular physical activity. College students represent a population at a pivotal stage of physical, psychological, and social development. This period is particularly sensitive for the establishment of long-term health behavior habits. While this group possesses unique advantages, such as strong autonomy and a high learning capacity, that support the adoption of healthy behaviors, they also encounter numerous challenges. These include academic pressures, career-related anxiety, adjustment to independent living, and the restructuring of social networks, all of which can undermine their exercise intentions and behaviors. Consequently, such factors may hinder the consistency and long-term stability of their health-related practices.

### 2.2 The perspective of theory of planned behavior

The theory of planned behavior is regarded as one of the most concise and predictive behavioral causal models in the fields of health psychology and health communication research, demonstrating considerable theoretical vitality over the subsequent decades (29). The theory suggests that an individual's intention to perform a behavior is the key proximal determinant of the actual behavior (30). Within the framework of the theory of planned behavior, the key variables include the individual's attitude toward a specific behavior, subjective norms, and the perceived ease or difficulty of performing the behavior. Thus, Based on the theory of planned behavior, it can be assumed that the more positive college students' attitudes toward physical activity are, the stronger the subjective norms, and the greater the perceived behavioral control, the stronger their intention to engage in physical activity, and the higher the likelihood of actually participating in such activities. However, despite some students expressing strong intentions to exercise, they ultimately fail to translate these intentions into actual behavior due to factors such as poor time management, lack of willpower, or insufficient external support.

## 3 Preliminaries

### 3.1 Machine learning evaluation metrics

Machine learning models have been widely applied in various health-related research fields with the continuous development of computer science. For example, Yan et al. (31) developed a lung cancer diagnostic model using SVM. Roitman et al. (32) employed machine learning to effectively distinguish between healthy and hoarse voices. Khalid et al. (33) reliably estimated the vascular age of healthy individuals by RF. Based on the above applications, the predictive performance of machine learning models is typically evaluated using metrics such as Accuracy, Precision, Recall, and F1-Score. The corresponding calculation formulas are as follows:

- Accuracy: Measures the overall proportion of correct predictions.

(1) $$\begin{array}{l} Accuracy=\frac{TP+TN}{TP+TN+FP+FN} \end{array}$$

Where *TP* (True Positive), *TN* (True Negative), *FP* (False Positive), and *FN* (False Negative) represent the classification outcomes.

- Precision: Indicates the proportion of correctly predicted positive cases among all predicted positives.

(2) $$\begin{array}{l} Precision=\frac{TP}{TP+FP} \end{array}$$

- Recall: Represents the proportion of actual positive cases that are correctly identified.

(3) $$\begin{array}{l} Recall=\frac{TP}{TP+FN} \end{array}$$

- F1-Score: The harmonic mean of Precision and Recall, providing a balanced measure especially useful for imbalanced datasets.

(4) $$\begin{array}{l} F1\text{-}Score=\frac{2\times \text{Precision}\times \text{Recall}}{\text{Precision}+\text{Recall}} \end{array}$$

### 3.2 SHAP explanation

The SHAP values can be used to explain the importance of different features in machine learning (34). According to (35), the calculation of SHAP values can be expressed as:


(5) $$\begin{array}{l} \phi_{i}=\underset{S\subseteq N\backslash \left\{i\right\}}{\sum }\frac{|S|!\left(|N|-|S|-1\right)!}{|N|!}\left[f\left(S\cup \left\{i\right\}\right)-f\left(S\right)\right] \end{array}$$

Explanation of the SHAP formula symbols as given in Table 1. In the SHAP method, each feature is assigned a SHAP value. The average SHAP value of a specific feature across all samples is considered as the contribution of that feature to the prediction outcome. Therefore, by introducing SHAP, the predictive logic of the exercise intention-behavior gap among college students can be explained.

**Table 1:** Explanation of SHAP formula symbols.

**Symbol**	**Description**
SHAP(*x*_*i*_)	Contribution of feature *x*_*i*_ to the prediction for a specific instance.
*N*	Set of all features, including *x*_*i*_.
*S*	Subset of features, excluding *x*_*i*_.
*f*(*S*)	Model's prediction when using the subset of features *S*.
|*S*|	Number of features in subset *S*.
|*N*|	Number of features in *N*.

## 4 Method

### 4.1 Data acquisition

In recent years, TikTok has garnered significant attention due to its large user base and high user engagement, particularly among college students. Therefore, we conducted an online questionnaire survey via the TikTok platform to collect data from university students aged 18 and above. To ensure data quality and relevance, participants were required to be current full-time undergraduate students and to complete all questionnaire items. A total of 2,000 responses were collected, of which 1,866 were deemed valid after excluding incomplete or inconsistent entries. The sample included students from different provinces across China, covering a variety of disciplines and university types, which enhances the representativeness of the data. Among the 1,866 valid responses, a small number of missing values are identified in individual variables. These missing values are handled using mean imputation to ensure data completeness and minimize potential bias.

### 4.2 Data preprocessing for exercise intention-behavior gap

Based on research by other scholars on the intention-behavior gap, the exercise gap in college students can be defined as the failure to meet the recommended physical activity standards despite having a strong intention to exercise or having a strong intention to exercise while meeting the recommended physical activity standards. In machine learning tasks, the intention-behavior gap can be treated as a binary classification label in supervised learning. According to the World Health Organization's "Physical Activity Guidelines," adults over the age of 18 should engage in at least 150 minutes of moderate-intensity physical activity per week. The research results show that among 1,866 college students with strong exercise intentions, approximately 56.27% exhibited the intention-behavior gap, while 43.73% did not. This phenomenon indicates that, despite most college students having strong intentions to exercise, there is still a significant gap in actual behavior. Specifically, more than half of the students, despite having strong exercise intentions, fail to meet the physical activity standards recommended by the World Health Organization.

### 4.3 Basic feature processing

To ensure a comprehensive analysis of the sample characteristics, we collected two basic demographic variables of college students: grade level and gender, which were used as feature variables in the machine learning models. According to the survey sample from TikTok, the results show significant differences in the exercise intention-behavior gap across different grade levels. Specifically, the largest number of students experiencing the gap are first-year students. This phenomenon can be attributed to the fact that first-year students are still adjusting to the new university environment. In contrast, senior students may experience a smaller intention-behavior gap due to greater autonomy, better time management, and higher levels of self-regulation developed through years of academic and social adjustment. They are also more familiar with available campus resources for physical activity, which can facilitate translating intention into action. Additionally, the proportion of females in the exercise intention-behavior gap is significantly higher than that of males. This disparity may stem from the fact that males generally have higher levels of interest in sports, competitive motivation, and exercise self-efficacy, making them more likely to actively engage in various forms of physical exercise.

### 4.4 Feature processing for health belief

Health belief refers to the psychological foundation upon which individuals base their engagement in health-promoting behaviors. This encompasses their cognitive and evaluative processes regarding health status, health risks, the effectiveness of health behaviors, and the feasibility of adopting such behaviors. Health beliefs among college students significantly influence their attitudes and decisions toward health behaviors. For instance, students with a higher awareness of health risks are more likely to prioritize health-promoting behaviors, thereby increasing their likelihood of engaging in proactive health actions. Moreover, self-efficacy—the belief in one's ability to successfully perform a specific health behavior—plays a crucial role within the context of health beliefs. The stronger a student's self-efficacy, the more likely they are to initiate and sustain health behaviors. Consequently, the perceived dimensions of health beliefs can be categorized into five key areas: perceived benefits, perceived barriers, perceived severity, self-efficacy, and perceived susceptibility. The specific options for the variables in each of these dimensions are outlined in Table 2.

**Table 2:** Health belief perceptual features.

**Feature**	**Sub-feature**
Perceived benefit (PBe)	1. Exercise is beneficial for health
	2. Exercise can help prevent diseases
Perceived barrier (PBa)	1. No suitable exercise facilities nearby
	2. Unable to find a suitable exercise method
Perceived severity (PSe)	1. Lack of exercise will lead to weight gain
	2. Lack of exercise will lead to low energy
Self-efficacy (SEf)	1. Exercise can be done every day
	2. Overcome the difficulties encountered during exercise
Perceived susceptibility (PSu)	1. The daily amount of exercise meets the standard
	2. Not engaging in proper exercise will accelerate aging

### 4.5 Feature processing for planned behavior

According to the Theory of Planned Behavior, an individual's rational behavior is driven by a series of perceived conceptual variables, including affective attitude, perceived subjective norms, and perceived behavioral control. Affective attitude reflects the individual's positive or negative evaluation of a certain behavior, often manifested through emotional responses. Perceived subjective norms refer to the social pressure or expectations from others that individuals perceive when performing a behavior, which can influence whether they choose to engage in a particular behavior. Perceived behavioral control refers to the individual's cognition about whether they have sufficient resources, abilities, or opportunities to perform a behavior, which directly influences their behavioral intention and actual behavior. The specific options for setting the characteristics of the variables in the planned behavior perception are shown in Table 3.

**Table 3:** Planned behavior perceptual features.

**Feature**	**Sub-feature**
Affective attitude (AAt)	1. A moderate level of exercise is beneficial for me
	2. A moderate level of exercise is enjoyable for me
	3. A moderate level of exercise is easy for me
Subjective norm (SNo)	1. My parents believe that exercise appropriately every day is necessary
	2. My doctor believes that I must exercise appropriately every day
	3. My partner believes that I must exercise appropriately every day
	4. I want to exercise according to my parents' wishes
	5. I want to exercise according to my doctor's advice
	6. I want to exercise according to my partner's wishes
Perceived behavioral control (PBC)	1. I can control my daily exercise level
	2. Proper exercise entirely depends on my willingness

### 4.6 Analysis of the reliability and validity of perceived features

To assess whether the characteristics derived from the questionnaire settings align with the expected structure, confirmatory factor analysis is conducted on the health belief perception and planned behavior perception characteristics, with the results presented in Table 4. According to the criteria (36) that the composite reliability (CR) of sub-factors within the same characteristic should exceed 0.7, or the average variance extracted (AVE) should be greater than 0.5, the extraction quality of the measurement indicators for each characteristic is deemed satisfactory. This indicates that the data structure of the characteristics aligns with the design expectations of the questionnaire. Furthermore, discriminant validity tests are performed to examine the correlations among the characteristics. The results of these tests are shown in Table 5. The findings reveal significant correlations among the characteristics (*P* < 0.001), and the absolute values of the correlation coefficients between all characteristics are smaller than the square roots of their respective AVEs. This suggests that there is sufficient differentiation among the characteristics, demonstrating good discriminant validity of the scale.

**Table 4:** Result of the validation factor analysis.

**Feature**	**Sub-feature**	**AVE**	**CR**
PBe	PBe1	0.525	0.677
	PBe2		
PBa	PBa1	0.530	0.685
	PBa2		
PSe	PSe1	0.533	0.694
	PSe2		
SEf	SEf1	0.538	0.732
SEf2			
PSu	PSu1	0.522	0.672
PSu2			
AAt	AAt1	0.642	0.819
	AAt2		
	AAt3		
SNo	SNo1	0.511	0.862
	SNo2		
	SNo3		
	SNo4		
	SNo5		
	SNo6		
PBC	PBC1	0.586	0.771
	PBC2		

**Table 5:** Results of the discriminant validity test, where 3*** represents 0.003*** and 0*** denotes 0.000***.

	**PBe**	**PBa**	**PSe**	**SEf**	**PSu**
PBe	0.717				
PBa	−0.062(3***)	0.809			
PSe	0.354(0***)	0.237(0***)	0.728		
SEf	0.263(0***)	0.091(0***)	0.366(0***)	0.517	
PSu	0.144(0***)	0.224(0***)	0.329(0***)	0.554(0***)	0.519
	**AAt**	**SNo**	**PBC**		
AAt	0.807				
SNo	0.172(0***)	0.685			
PBC	0.299(0***)	0.469(0***)	0.531		

### 4.7 Model construction

First, the 1866 data points are split into training and testing sets in a 3:1 ratio, with 23-dimensional features used as inputs. Three machine learning models are constructed: LR, SVM, and RF. Next, by selecting the optimal model, the aim is to identify whether there is a gap between college students' exercise willingness and behavior. To improve model performance, grid search and five-fold cross-validation are used to optimize the parameters of each model. Model evaluation metrics include accuracy, precision, recall, and F1-score. Finally, the features that contribute the most to the exercise intention-behavior gap in the models are analyzed based on the SHAP method, providing theoretical support and practical guidance for subsequent research.

### 4.8 Model configuration

The main hyperparameters for the machine learning models used in this study are determined via grid search combined with five-fold cross-validation to ensure optimal performance. Specifically, the Support Vector Machine (SVM) employs an RBF kernel with a penalty parameter *C* = 0.5 and a kernel coefficient


$$\gamma =\frac{1}{\text{number of features}}.$$

The Random Forest model utilizes 100 trees with a maximum depth of 30. The Logistic Regression model is configured with the lbfgs solver, a maximum of 100 iterations, and an L2 regularization penalty. All experiments are conducted on a computing platform equipped with an Intel Xeon Gold 5218 CPU (16 cores), an NVIDIA Tesla V100 GPU (32 GB memory), and 128 GB of RAM, running Ubuntu 20.04 LTS. The Python environment used is version 3.8. This setup guarantees the reproducibility and reliability of the experimental results.

## 5 Result analysis

### 5.1 Prediction result analysis

**Figure 2:**
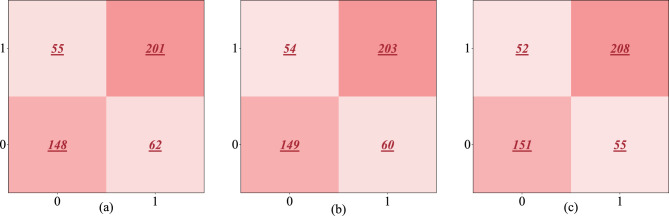
Confusion matrices of predictions from three machine learning models. **(a)** LR. **(b)** SVM. **(c)** RF.

To intuitively demonstrate the classification performance of the different models in predicting the intention-behavior gap in college students exercise behavior, we plot the confusion matrices corresponding to each model, as given in Figure 2. In these matrices, 0 represents the absence of the intention-behavior gap, while 1 indicates its presence. The color intensity reflects the number of predicted samples, with darker shades indicating a higher quantity. As shown in Figure 2, the three machine learning models effectively distinguish between the presence and absence of the exercise intention-behavior gap. This result indicates that AI-based modeling approaches successfully extract the underlying decision-making logic between exercise intention and behavior among college students by capturing multivariable interactions and the overall data structure.

**Figure 3:**
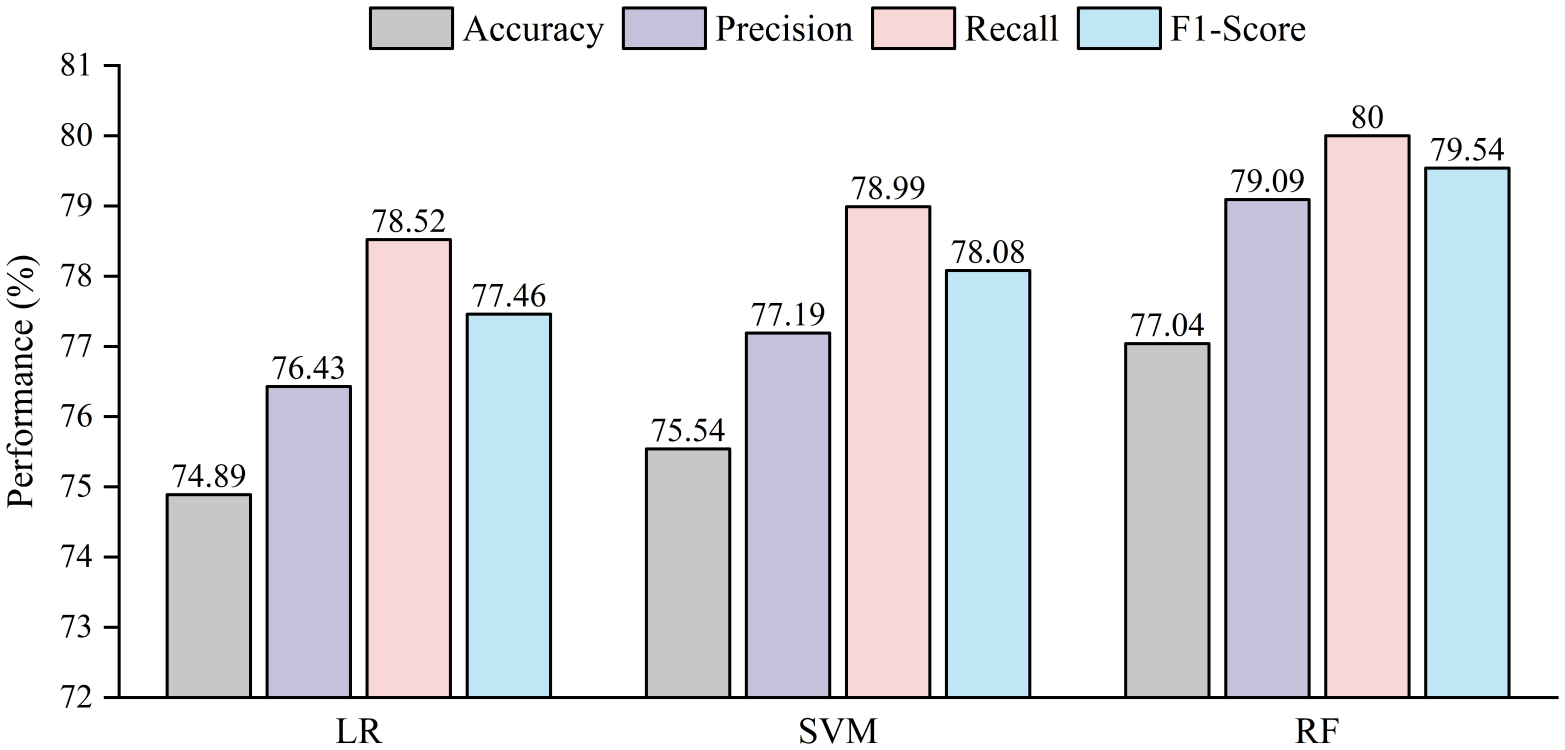
Comparison of the performance of the models. **(a)** LR. **(b)** SVM. **(c)** RF.

In addition, Figure 3 further presents the performance of the three models in this prediction task, including accuracy, precision, recall, and F1-score. The results show that LR performs the worst, with an accuracy of 74.89%, precision of 76.43%, recall of 78.52%, and F1-score of 77.46%, indicating that the factors influencing the intention-behavior gap in college students exercise behavior exhibit strong nonlinearity. SVM performs better than LR, achieving an accuracy of 75.54%, precision of 77.19%, recall of 78.99%, and F1-score of 78.08%, suggesting its ability to capture certain nonlinear patterns in the data. Notably, RF achieves the best classification performance, with an accuracy of 77.04%, precision of 79.09%, recall of 80%, and F1-score of 79.54%, demonstrating its superior capacity to handle complex variable interactions. In summary, machine learning methods based on artificial intelligence, especially the RF model, demonstrate strong potential for predicting the intention-behavior gap in college students exercise behavior.

### 5.2 Feature importance analysis

**Figure 4:**
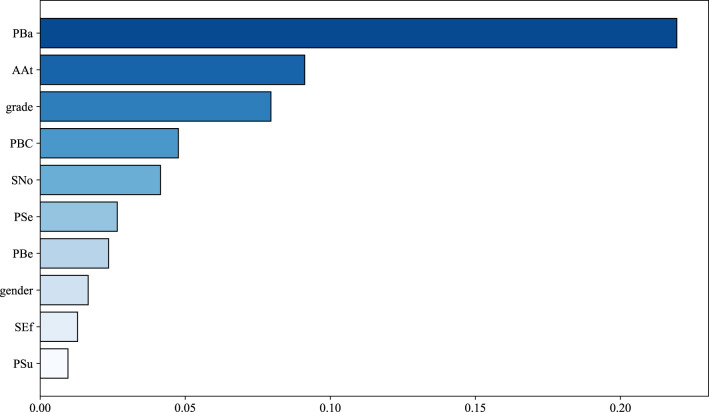
Feature bar chart based on the SHAP method.

To investigate the contribution of each feature variable to the intention-behavior gap between college students exercise intentions and their actual behaviors, a SHAP-based bar chart of feature importance is constructed, as shown in Figure 4. Specifically, the average absolute SHAP values across all samples are computed to quantify the influence of each feature on the model's output. As illustrated in Figure 4, the features are ranked in descending order of importance as follows: PBa, AAt, grade, PBC, SNo, Pse, PBe, gender, SEf, and PSu. Among these, PBa exhibits the greatest influence on the intention-behavior gap, while PSu shows the least impact.

**Figure 5:**
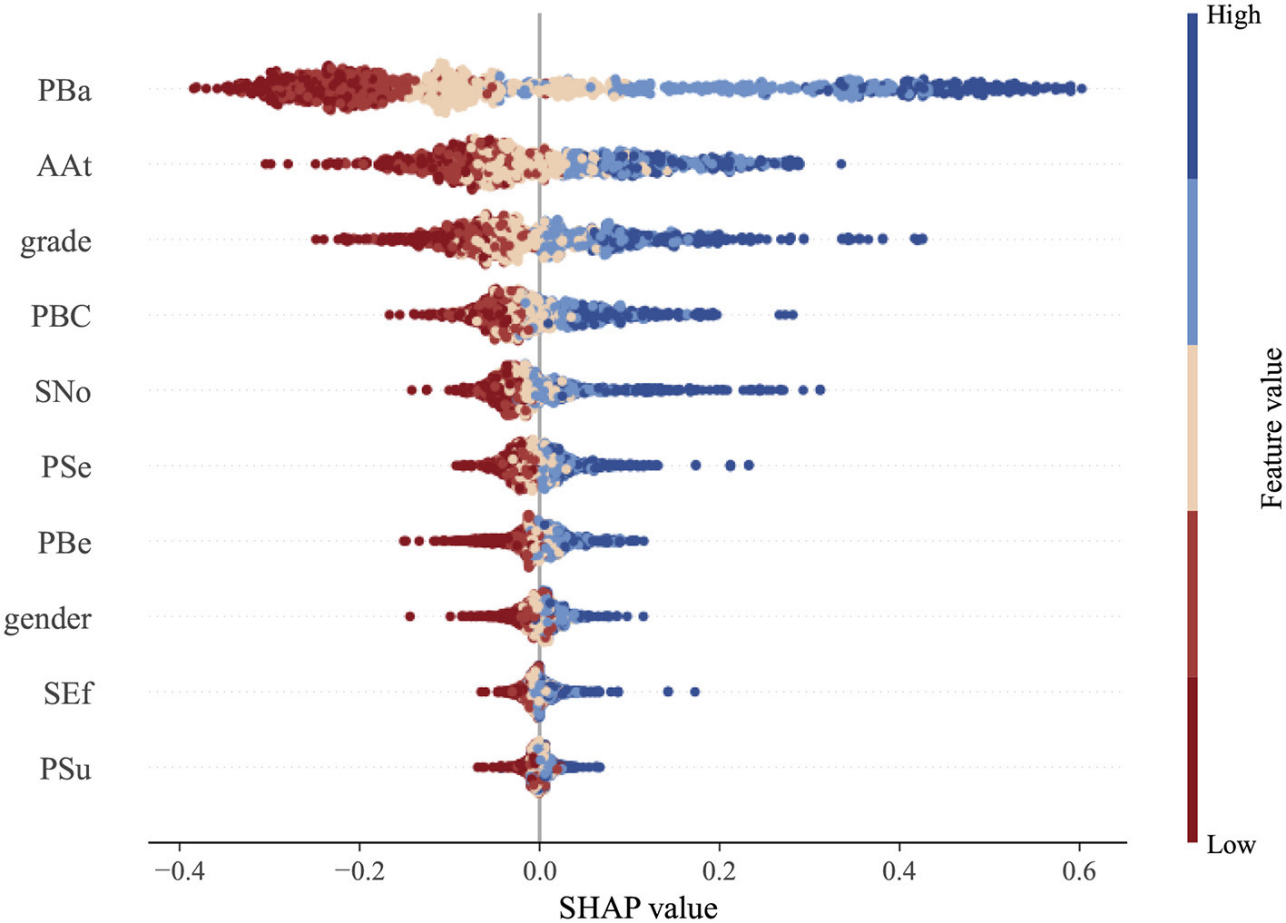
Feature summary plot based on the SHAP method.

To further interpret the overall influence of each feature on the intention-behavior gap, a SHAP summary plot is generated, as shown in Figure 5. In this plot, the SHAP value greater than 0 indicate a narrowing of the gap, while value less than 0 suggest a widening. From Figure 5, increases in health belief perception and perceived behavioral control are generally associated with higher SHAP value, indicating that these factors contribute to reducing the gap between intention and behavior. This observation aligns with findings from previous studies.

It is important to highlight that the impact of the grade in our study diverges from previous research. Our findings indicate that the intention-behavior gap diminishes as grade level increases. While earlier studies suggest that freshmen face challenges in adapting to their new environment, and seniors are constrained by pressures related to internships and job hunting, limiting their time and energy for physical activity, our results suggest a shift in this trend. With societal advancements and an increasing awareness of health benefits, more college students now recognize that regular physical activity not only enhances physical health but also alleviates stress. This recognition has fostered a more consistent engagement in exercise routines. Additionally, the SHAP value for the variables SEf and PSu are primarily clustered around zero, indicating that these variables exert minimal influence on the intention-behavior gap.

### 5.3 Analysis of the impact of feature on the prediction path

**Figure 6:**
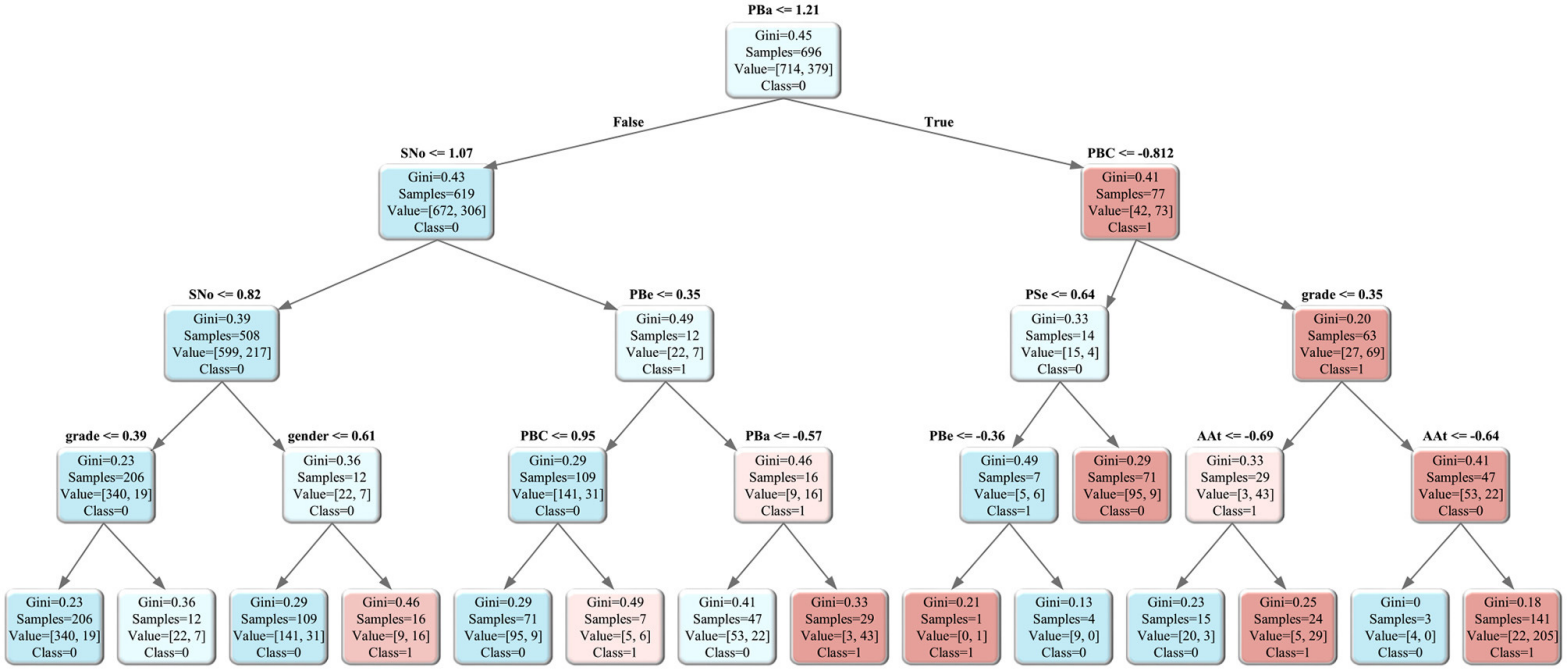
Decision tree of prediction path.

Based on the analysis of key feature variables, a decision tree model is further plotted to illustrate the role of these features in the exercise behavior transformation path, as shown in Figure 6. We tune the decision tree's parameters and find that a maximum depth of 4 best captures the key features identified in the feature importance analysis, which achieves the highest consistency between tree nodes and important variables. Therefore, a maximum depth of 4 is selected as the final parameter for plotting. Under this setting, the Gini index-based partitioning results reveal that PBa is identified as the root node, with PBC and SNo serving as key child nodes. As shown in Figure 6, college students first consider perceived barriers to exercise when making decisions about exercise behavior. When perceived barriers are high, individuals tend to further refer to perceived subjective norms; however, when perceived barriers are low, the likelihood of an exercise intention-behavior gap decreases. Furthermore, if perceived behavioral control is weak, the probability of high-grade male students experiencing an intention-behavior gap in exercise is reduced. Overall, perceived barriers to exercise emerge as the primary predictor of the intention-behavior gap, while perceived behavioral control plays a crucial role in reducing this gap.

## 6 Conclusion

In this article, to accurately predict the intention-behavior gap in exercise among college students, we construct multiple machine learning models, including Logistic Regression (LR), Support Vector Machine (SVM), and Random Forest (RF). By comparing the performance of different models, the best-performing RF model is selected, and the SHAP method is introduced for interpretability analysis. This analysis reveals the key feature variables that influence the gap between college students' exercise intentions and actual behaviors, providing intervention priorities and directions for narrowing the gap between exercise intentions and behaviors in real-world contexts. In addition, we introduce variables such as health belief perception and planned behavior perception into the research framework of the exercise intention-behavior gap, examining the multidimensional factors influencing this gap from a more holistic and systematic perspective, which has theoretical significance for the prediction and analysis of the influencing factors of the exercise intention-behavior gap. Based on the conclusions, the following recommendations for addressing the exercise intention-behavior gap among college students are proposed:

1. Perceived barriers are a key factor influencing the existence of the intention-behavior gap in college students' exercise willingness. The ability of students with strong intentions to exercise to engage in physical activity and meet the recommended standards of physical activity largely depends on their perceptions of barriers to exercise behavior. Therefore, universities' efforts to promote and intervene in physical activity should begin by addressing and removing these perceived barriers. Specifically, universities should strategically allocate and optimize public sports resources, ensuring that students have access to sufficient exercise facilities. Moreover, universities should actively foster a supportive sports culture, which includes widespread dissemination of fundamental sports knowledge and the enrichment of physical education curricula. On this foundation, enhancing students' emotional attitudes toward the lack of physical activity will further facilitate the elimination of the intention-behavior gap.
2. Based on the explainable machine learning model, both grade level and gender exhibit a significant predictive effect on the intention-behavior gap among university students. The variations observed across different grade levels reflect the distinct challenges students face at various stages of their academic journey, including time pressures, shifting life rhythms, and evolving health awareness. These factors may influence students' motivation and capacity to engage in physical exercise. Therefore, it is essential for universities to extensively promote the benefits of physical activity for both physical and mental health. Thus, universities should widely promote the positive impact of physical exercise on physical and mental health, and enhance students' awareness of physical education by offering physical fitness development courses. Furthermore, considering gender differences, universities should place a strong emphasis on ensuring the accessibility of sports participation for female students in the planning and management of sports facilities. This approach is crucial for enhancing their willingness to engage in physical exercise and increasing their overall participation in sports activities.
3. Based on the feature importance ranking of the model and the decision tree analysis, it is clear that perceived barriers play a primary role in predicting the exercise intention-behavior gap among university students. In contrast, the effects of emotional attitude, perceived benefits, and other feature variables appear to be less pronounced. Therefore, further research is needed to investigate the moderating effects of perceived barriers, emotional attitude, and perceived benefits on the relationship between exercise intention and behavior in university students.

## 7 Limitations

The data samples in this study are primarily collected from university students in China. Due to the relatively homogeneous regional and cultural background, the generalizability of the findings may be limited across the entire country or other cultural contexts. Variations in economic development, educational resources, and social culture across different areas in China may influence the intention-behavior gap in physical exercise among university students differently. In future work, we aim to expand the sample coverage to include a broader range of regions and types of universities globally to validate the model's generalizability and robustness. Moreover, factors such as urban-rural differences, economic conditions, and cultural backgrounds will be further explored to understand their impact mechanisms on students exercise intentions and behaviors. Additionally, longitudinal studies considering dynamic changes over time will be conducted to enhance the model's adaptability to real-world environments.

## Data Availability

The original contributions presented in the study are included in the article/supplementary material, further inquiries can be directed to the corresponding author.
